# WSES guidelines on blunt and penetrating bowel injury: diagnosis, investigations, and treatment

**DOI:** 10.1186/s13017-022-00418-y

**Published:** 2022-03-04

**Authors:** Luke Smyth, Cino Bendinelli, Nicholas Lee, Matthew G. Reeds, Eu Jhin Loh, Francesco Amico, Zsolt J. Balogh, Salomone Di Saverio, Dieter Weber, Richard Peter ten Broek, Fikri M. Abu-Zidan, Giampiero Campanelli, Solomon Gurmu Beka, Massimo Chiarugi, Vishal G. Shelat, Edward Tan, Ernest Moore, Luigi Bonavina, Rifat Latifi, Andreas Hecker, Jim Khan, Raul Coimbra, Giovanni D. Tebala, Kjetil Søreide, Imtiaz Wani, Kenji Inaba, Andrew W. Kirkpatrick, Kaoru Koike, Gabriele Sganga, Walter L. Biffl, Osvaldo Chiara, Thomas M. Scalea, Gustavo P. Fraga, Andrew B. Peitzman, Fausto Catena

**Affiliations:** John Hunter Hospital, University of Newcastle, Newcastle, NSW Australia

**Keywords:** Penetrating trauma, Blunt trauma, Bowel injury, Bowel trauma, Bowel injury diagnosis, CT signs bowel injury, CT diagnosis bowel injury, Bowel injury management, Surgical management bowel injury

## Abstract

The aim of this paper was to review the recent literature to create recommendations for the day-to-day diagnosis and surgical management of small bowel and colon injuries. Where knowledge gaps were identified, expert consensus was pursued during the 8th International Congress of the World Society of Emergency Surgery Annual (September 2021, Edinburgh). This process also aimed to guide future research.

## Background

Traumatic hollow viscus and mesenteric injury are relatively uncommon, with a prevalence of approximately 1% in blunt trauma and 17% in penetrating trauma [1]. Following blunt and penetrating trauma, and especially in the context of multiple other injuries, hollow viscus and mesenteric injuries pose a clinical challenge mainly due to their relative infrequency, diagnostic uncertainties, and deleterious consequences when not promptly treated.

The aim of this paper was to review the recent literature, to create recommendations for the day-to-day diagnosis and surgical management of small bowel and colon injuries. Using GRADE methodology [2] for evidence evaluation and grading, a literature review was conducted. The expert consensus occurred during the 8th International Congress of the World Society of Emergency Surgery (September 2021, Edinburgh) on the following topics: diagnosis of bowel injuries, role, and pitfalls of CT in the diagnosis of bowel injury; peritoneal lavage; diagnostic laparoscopy and therapeutic laparoscopy; damage control versus definitive management; handsewn versus stapled anastomosis; missed bowel injury management and outcomes.

## Blunt abdominal trauma: observation and nonoperative management

The clinical assessment for a patient with suspected intestinal injury begins with the primary survey to assess life-threatening injuries. Patients who are haemodynamically decompensated with a positive Focused Assessment with Sonography for Trauma (FAST) should proceed directly to a trauma laparotomy to stop major abdominal bleeding and, if applicable, other sources of bleeding (e.g., pelvic, or long bone fractures), as well as control spillage of intestinal contents. In circumstances where the degree of haemodynamic decompensation is minor, diagnostic, and therapeutic laparoscopy can be considered. It is important to emphasise that these considerations are time crucial, with every 3 min spent in the emergency department equating to a 1% increased death probability [3] and as a result of such, trauma laparotomies tend to be favoured.

In all trauma patients it is important to first identify the mechanism of injury. The abdomen should then be inspected for evidence of penetrating injury/impalement, distension, asymmetry, lacerations, abrasions, and other blunt forces such as a “seatbelt sign” to alert of a possible intra-abdominal injury. Indeed, patients with blunt abdominal injury may not have any external signs of trauma. The initial clinical assessment may be difficult and inaccurate due to distracting injuries; associated injuries of the abdominal wall, rib cage and pelvic girdle mimicking signs of guarding, or other injuries which mask pain, such as head and spinal cord injuries. Significant abdominal tenderness on palpation and involuntary guarding are signs of peritonitis and are suggestive of leakage of intestinal contents but may take several hours to develop. Peritoneal signs tend to develop slowly in small bowel injury as luminal contents have a neutral pH, are enzymatically less active and have a relatively low bacterial load. Furthermore, bowel perforation can be a delayed response because of vascular injury resulting in bowel ischemia and necrosis, as such peritoneal signs may take many hours to develop. Accuracy increases if the patient has serial examinations as part of non-operative management. FAST scan can identify varying levels of free-fluid and is highly operator dependent. On average 620 mL is required to be detected but in the hands of a highly skilled operator (top 10%) as little as 400 mLs can be detected [4]. FAST scan improves as it is repeated, allowing for more time for fluid to accumulate. Intra-abdominal free fluid on FAST is non-specific for intestinal injury and should not be relied upon in these settings to diagnose bowel trauma [5, 6]. Furthermore FAST scan can be utilised to detect free intraperitoneal air in the hands of an experienced sonographer, this free air may be an indication of bowel perforation [7]. Intravenous contrast enhanced computed tomography (CT) scan may identify intra-abdominal injuries, but bowel injury remains one of the most common abdomino-pelvic injury missed on initial CT at 20% of bowel injuries missed [8]. Patients who have equivocal or non-specific findings on the initial CT should be admitted for observation for potential intestinal injury. Observed patients require close monitoring and serial clinical examination. Observation in patients with high-risk mechanism, clinical or radiological suspicion or in comatose state might include a repeated CT scan. In patients with equivocal initial CT findings this repeat scan should take place after 6 h. In patients with evolving clinical features that increase suspicion, yet not enough to warrant surgery, a repeat CT scan should be strongly considered. If a polytrauma patient with critical injuries in other systems (severe brain injury, multiple long bone fractures) is already on the operating table and unlikely to be clinically assessed postoperatively, the benefit from abdominal exploration may outweigh the risk of a missed injury. Diagnostic laparoscopy and diagnostic peritoneal lavage (DPL) are alternatives. Any deteriorating patient should be taken for exploratory surgery [9].

Seatbelt syndrome is a term coined by Stassen et al. [10] but recognised decades prior [11], which describes a group of injuries that occur during a motor vehicle accident due to the impact of vehicle restraints on a person’s torso. This syndrome was particularly notorious during the era of two-point seat belts. The seatbelt syndrome includes: lumbar spine fracture, bowel perforation and a positive seatbelt sign [12]. A positive seatbelt sign (namely bruised skin where the seat belt has restrained the passenger) is linked to a 12% chance of bowel injury [13]. The most common injuries identified intraoperatively in patients with seatbelt sign involve small bowel (58%), large bowel (39%), and the spleen (39%) [14]. Seatbelt sign should prompt definitive imaging with CT scan and the subgroup of patients with negative CT scan should be admitted for serial examination [14, 15]. Those patients with a positive CT and seatbelt sign should be evaluated for operative or non-operative management. Similar injuries may be identified in paediatric populations from the impact of the handlebar of a bicycle, in such situations the same clinical suspicion should be applied [16].

Serum procalcitonin, rather than white cell count (WCC), CRP, or IL-6, has been suggested as a useful indicator of bowel injury in polytrauma patients. Procalcitonin was shown to be increased in the first two days following liver and bowel injury when compared with other abdominal injuries (spleen, mesentery, retroperitoneum) or in patients without abdominal injury [17]. CRP instead raises in almost all trauma patients regardless of an abdominal injury, with a longer time to peak, and was not found to be influenced by the presence of bowel injury, hence not useful as a biomarker for bowel trauma. Any biomarker should be used in conjunction with clinical and radiological assessment rather than an independent predictor of abdominal injury to avoid unnecessary surgical management [17–19]. Emerging monitoring methods such as the compensatory reserve measurement, which when compared to CRP, vital signs, and quick sequential organ failure assessment (qSOFA) score, demonstrate a higher sensitivity for sepsis or septic shock and may play a crucial role during the observation of patients at a higher risk for bowel injuries based on mechanism of injury or CT finings [20]. These clinical adjuncts can be utilised to diagnose bowel injury in unconscious patients. It must be highlighted that these become progressively more accurate over time by analysing the trend rather than the initial value (which is often raised in all seriously injured trauma patients). Therefore, it is crucial to repeat these tests, serially whilst they’re an inpatient and not disregard test values based upon initial numbers. The frequency of repeating tests will vary largely on clinical suspicion, clinical examinations may occur as frequently as 8 hourly, whereas blood tests may be repeated every 24 h. In unconscious patients, at risk of but without definitive evidence of bowel injury, tolerance to enteral feeding might be used as negative predictor of bowel injury. Tolerance of enteral feeding is greatly influenced by gut motility [21], which may be a subtle indicator of traumatic injury to the small bowel or colon [22]. Experienced clinicians will wait until the probability of bowel injury is low before commencing enteral feeding, as such, feeding should not be used with the intent to discover bowel injury, but failure to tolerate feeding should raise clinical concern. A mixed methodology study indicated 15% of ICU patients who failed enteral feeding had gastrointestinal injury [23]. Failed enteral feeding has also been identified in 147 ICU patients to be linked to higher rates of sepsis, readmission to ICU and longer ICU stays (*p* = 0.007, *p* = 0.04 and *p* = 0.001), thus the failure of enteral feeding is a poor prognostic factor [24].

**Recommendations**

1. Management of the awake and oriented blunt abdominal trauma patient starts with the primary survey, E-FAST, physical examination and the secondary survey, blood chemistry, vital signs followed by contrast-enhanced abdominal CT. **(GRADE: High)**
2. The presence of a seatbelt sign should prompt a CT scan and a high index of suspicion for bowel injury. **(GRADE: High)**
3. Patients with high-risk mechanisms (i.e., handlebar, seatbelt sign) and non-specific CT findings should be admitted for observation including serial clinical examination. **(GRADE: Moderate)**
4. In patients not clinically evaluable, the diagnosis of hollow viscus injuries relies on injury pattern, vital signs, inflammatory markers trends and follow-up CT. **(GRADE: Moderate)**
5. In selected cases a repeat CT might be considered. Patients with equivocal signs on initial CT scan should be re-imaged after 6 hours. Patients that demonstrate evolving clinical signs suspicious for bowel injury, re-imaging should be considered. **(GRADE: High)**
6. Although highly sensitive, serum procalcitonin and CRP are not necessarily specific and as supportive biomarkers will help to exclude bowel injuries; but if too heavily relied upon, may lead to nontherapeutic laparotomy, or missed bowel injury. **(GRADE: Moderate)**

## Blunt abdominal trauma: role and pitfalls of CT in the diagnosis of bowel injury

Modern trauma care substantially relies on E-FAST [25] and CT for blunt trauma patient management. However, to diagnose bowel injuries, the role and diagnostic accuracy of CT remains low. Radiological signs that are sensitive rarely hold much weight in management when alone, and those signs that are specific for bowel injury rarely occur [26–36]. Much of the research regarding sensitivity and specificity of bowel injury though is based on data obtained through lesser quality CT scanners which may have improved in quality in recent years. A recent literature review combining all the data sets published between 1990 and 2015 evaluated the largest population group presented so far (11,924 patients) [29]. Of this group, the most sensitive sign was free peritoneal fluid. When the free fluid was detected without solid organ injury, it carried less sensitivity but higher specificity (53% and 81%, respectively). Bowel wall thickening and mesenteric stranding had the next highest sensitivities. Highly specific signs for bowel injury include bowel wall hematoma, oral contrast extravasation, and the presence of free intraperitoneal air without pneumothorax (Table 1). It must be noted that free intra-abdominal air can be a result of air tracking from a pneumothorax through the crura of the diaphragm causing a false positive result [37].

**Table 1:** Performance of CT in detecting bowel injury requiring surgical treatment on 11,924 blunt trauma patients [29]

CT sign	Sensitivity (%)	Specificity (%)
Free fluid	66	85
Bowel wall thickening	35	95
Mesenteric stranding	34	92
Mesenteric hematoma	34	99
Bowel wall haematoma	23	100
Oral contrast extravasation	10	100
Bowel wall discontinuity	22	99
Intravenous contrast extravasated in mesentery	23	100
Free air	32	99
A wall enhancement	30	96

Scoring systems that combine CT findings have shown better performance for bowel injury detection. Faget’s scoring system was retrospectively and internally validated on 556 blunt trauma patients [38]. Their study further graded CT signs with scores associated with relevance (small haemoperitoneum scored 1 point, whereas pneumoperitoneum scored 5 points) (Table 2). Patients who scored five or higher had an 11-fold increase of bowel injury requiring surgical exploration (AUC was 0.98). Similarly, Bonomi and co-workers developed six criteria for detecting hollow viscus and mesenteric injury requiring surgical repair [1]. Their criteria were: free air, free fluid without solid organ injury, intra-mesenteric fluid, contrast extravasation (blush), bowel wall abnormality (including thickening) and mesenteric alteration (including stranding). In a cohort of 114 blunt trauma patients who underwent explorative laparotomy, four or more of the above CT findings were pathognomonic for a bowel injury requiring surgical treatment. Other researchers have added clinical parameters to these grading scales. McNutt and co-workers retrospectively reviewed a population of 110 blunt trauma patients [39]. They utilised a grading scale to evaluate the mesentery on CT (Table 3). A grade of four or higher and clinical findings such as abdominal tenderness or raised WCC conferred a higher risk of bowel injury (AUC, sensitivity, and specificity of 0.81, 86% and 76%, respectively)**.** Very recently, a prospective validation study by Wandling et al. confirmed the utility of McNutt’s score [40]. With a score of ≥ 2 patients were ten or more times likely to require a laparotomy (AUC, sensitivity, and specificity of 0.75, 72% and 78%).

**Table 2:** CT grading and indication for surgical exploration for possible bowel injury following blunt trauma developed by Faget et al. A score of 5 or more is diagnostic of bowel injury and prompt intervention [38]

CT Sign	Score
Haemoperitoneum, small	1
Haemoperitoneum, abundant	3
Mesenteric pneumoperitoneum	5
Bowel wall thickness	2
Arterial mesenteric vessel extravasation	3
Mesenteric stranding	2
Reduced bowel wall enhancement	1
Bowel wall discontinuity	5
Splenic injury	− 1
Anterior abdominal wall injury	2

**Table 3:** CT grading of mesenteric injury developed by McNutt et al. [39]

Grade	CT sign
1	Isolated mesenteric contusion
2	Mesenteric hematoma < 5 cm
3	Mesenteric haematoma > 5 cm
4	Mesenteric contusion or haematoma (any size) with bowel wall thickening and adjacent interloop fluid collection
5	Active vascular or oral contrast extravasation, bowel wall transection or pneumoperitoneum

In the context of high-risk mechanisms (MVA > 64kmph, pedestrian vs car, fall > 20 m, etc. [41]) without peritoneal signs but subtle signs on initial CT, a follow-up CT can improve sensitivity and specificity in detecting bowel injuries [42, 43]. With time enteric fluid pools and intraluminal gas can leak in the peritoneal cavity. Thus, the presence of free fluid or mesenteric standing on initial CT with or without solid organ injury should prompt a very high index of suspicion and a low threshold for a follow-up scan. Repeating CT scans is criticised due to increased ionizing radiation exposure, potential kidney damage, and minimal detection rate (the latest highly dependable on the threshold to repeat the scan) [44]. Despite the mixed evidence, there is certainly a subset of blunt trauma patients at risk for bowel injury for whom a follow-up CT will be crucial. A follow-up abdominal CT should certainly be considered in comatose polytrauma patients who require further imaging, such as a follow-up brain CT. Others have recommended repeating the CT scan if clinical improvement is not apparent within an 8-h window period. In contrast, a delay of 24 h will be even more likely to be diagnostic but linked to a higher complication rate and an increased overall mortality. [32].

**Recommendations**

7. The presence of highly specific CT findings such as extraluminal air, extraluminal oral contrast, or bowel-wall defects warrants prompt surgical exploration. **(GRADE: High)**
8. The presence of highly sensitive CT findings such as free fluid in the absence of solid organ injury, abnormal enhancement of bowel wall, and mesenteric stranding can be used as an adjunct to the clinical picture but should not solely determine management. **(GRADE: Moderate)**
9. Scoring systems that include radiologic, biochemical, and clinical signs can guide management in difficult scenarios. **(GRADE: Moderate)**
10. A repeat CT scan can be considered in patients with high-risk mechanisms without peritoneal signs and subtle signs on initial CT of bowel injury who do not show clinical improvement or are not clinically evaluable. **(GRADE: Moderate)**

## Penetrating abdominal trauma: observation and nonoperative management

Patients with penetrating abdominal trauma can be selected for nonoperative management (NOM) in certain civilian trauma centres. There is no standard generalizable classification; however there have been guidelines and algorithms proposed [6, 45, 46]. If there is no immediate indication for laparotomy a local wound exploration (LWE) is performed to rule out peritoneal violation. The utilisation of local wound exploration is only studied well with respect to anterior abdominal wounds, there is little evidence for gunshot wounds or flank and posterior wounds. The sensitivity of LWE varies with clinician experience, there is a small chance of false negative exploration in smaller stab wounds. If the injury does not breach the anterior fascia the patient can be discharged home (other injuries permitting). If the injury does breach the peritoneum non-operative management can still be utilised, however one should have a higher clinical suspicion for injuries on serial clinical examinations and a lower threshold for intervention [45, 47]. NOM is an option only when all the resources are available for: serial clinical examinations performed by experienced clinicians, vital signs monitoring, prompt access to the operating theatre, and ICU admission if required. Any decrease in haemoglobin concentration < 2 g/dL from baseline, or presumed, without other explanation than the penetrating abdominal wound [48], worsening vital signs or clinical examination should prompt surgical exploration [6, 49]. If NOM is elected, CT of chest, abdomen and pelvis can be a crucial adjunct. The choice to scan is highly dependent on the mechanism, location, depth, and number of wounds, and is not always required. Stab wounds to the back or flank rely upon CT to determine damage to retroperitoneal organs or the colon where clinical assessment may be challenging. Anterior stab wounds however can be more easily assessed, and CT scans should be less relied upon. A negative CT should not be used as the sole determinant for discharging a patient, unless a tangential and extraperitoneal wound tract is confirmed [50].

Most of the available literature and experience of NOM is for stab wounds. However recently this has also been applied to gunshot wounds (GSW). The reluctance for NOM in GSW is partly due to the irregular pattern of injury from GSW in terms of trajectory, cavitation, and the relatively high incidence of hollow visceral injury. Mandatory laparotomy has traditionally been considered for GSW to the abdomen, but a few centres have identified a subset of patients where NOM may be considered [51, 52]. These are haemodynamically compensated patients with no peritonitis or abdominal tenderness with a tangential injury and clear CT evidence of no intra-abdominal injury. In their study of 249 patients, Inaba et al. have shown CT to be inferior to clinical examination to detect the need for surgical intervention [53]. The specificity and sensitivity for bowel injury through clinical examination were 99% and 100%, respectively, as compared to 84% and 31% with CT [50]. A lower threshold to exploration should be employed in patients with GSW penetrating injuries, and a minimum of 48 h of observation must be performed [53].

Patients who are haemodynamically decompensated or have peritonitis are not candidate for NOM. Other “hard signs” to proceed to laparotomy include blood per rectum, enteric matter in the wound, impalement, organ evisceration, hematemesis, or frank blood on NG aspiration. Trauma patients with unreliable clinical examination (intubated, intoxicated, psychiatric illness) should not be candidates for NOM. [6, 49–53]. Patients who are managed with NOM can potentially be discharged after 48 h if clinically improving.

**Recommendations**

11. NOM can be performed at specialised centres in patients with penetrating abdominal trauma provided that the patient is haemodynamically compensated and cooperative. NOM might be more suitable for stab wounds when compared to GSW. **(GRADE: Moderate)**
12. When CT does not identify hard signs of bowel injury, LWE or screening laparoscopy to investigate for peritoneal violation will guide toward a laparotomy or NOM. Patients without peritoneal violation can be safely discharged. **(GRADE: Moderate)**
13. NOM requires at least 48 hours of serial clinical examinations, performed by consistent specialists or consultants, vital sign monitoring, and serial inflammatory markers testing. **(GRADE: Moderate)**

## Penetrating anterior abdominal injury: role and pitfalls of CT in the diagnosis of bowel injuries

The role of CT in managing anterior penetrating trauma patients is arguably more controversial when compared to blunt trauma. The clinical utility of screening, diagnostic or therapeutic laparoscopy in penetrating anterior abdominal trauma is proving to be a safer alternative [54]. Nevertheless, CT has a sensitivity of 88% and specificity of 72% for detecting bowel injury in penetrating trauma and remains the test of choice for flank and posterior entry wounds. The sensitivity of CT in detecting bowel injury is 88% and 80% for gunshot and stab wounds, respectively [55]. The radiological appearance of small bowel and colon injury following penetrating trauma on CT is much the same as blunt trauma. Free fluid is still the most common CT finding of bowel injury followed by mesenteric stranding, bowel wall thickening and then extravasation of contrast (whereas free air is expected in penetrating injury). Additionally, in the context of gunshot wounds, metallic fragments within the intestinal wall or lumen may be detectable and are of high specificity for bowel injury. Furthermore, in general gunshot wounds distribute high kinetic energy to the surrounding tissues compared to stab wounds [56]. This allows for easier interpretation on CT. Not every bullet and tissue interaction are the same however, due to tumbling, fragmentation, unimpeded passage, and the potential for cavitation [57]. As such gunshot wounds should all be treated individually and with caution. In the context of penetrating trauma, there was a preference for triple contrast CT (IV, rectal and oral) to better demonstrate extravasation of contrast. However, the increased sensitivity of newer multidetector CT scanners, long transit times of enteral contrast, and the comparable accuracy of single IV contrast scans to demonstrate bowel injuries have made single IV contrast the standard modality [55].

Centres that routinely explore haemodynamically compensated patients with penetrating abdominal injuries may still benefit from a preoperative CT to predict challenging liver, retroperitoneal and/or great vessels injuries and prepare the surgical team for such a scenario. That is not to say that every patient requiring surgery needs a pre-operative CT scan for planning of surgery, but rather the CT scan can be useful in certain situations. In circumstances where a patient may deteriorate in the CT scanner, an operation should not be delayed.

**Recommendations**

14. Following penetrating trauma, highly specific CT findings for bowel injury include extraluminal air, extraluminal contrast, bowel-wall defects and metallic fragments within the intestinal wall or lumen. **(GRADE: High)**
15. Following penetrating trauma, highly sensitive CT findings for bowel injury include free fluid in the absence of solid organ injury, abnormal enhancement of bowel wall and mesenteric stranding. These can be used as an adjunct in the clinical picture but should not solely determine management. **(GRADE: Moderate)**
16. IV contrast-enhancing CT scan has equal sensitivity to triple contrast in detecting bowel injury and is favourable in time-sensitive trauma situations. **(GRADE: Low)**
17. Serial clinical examinations are complementary to CT in guiding surgical management in trauma centres that practice the NOM approach in penetrating abdominal trauma. **(GRADE: Moderate)**

## Role of peritoneal lavage, diagnostic laparoscopy, and therapeutic laparoscopy

### Diagnostic peritoneal lavage

Peritoneal lavage, the technique of withdrawing peritoneal fluid and assessing the presence of frank blood was first described in 1965 [58] as an alternative to an exploratory laparotomy to diagnose intra-abdominal bleeding in haemodynamically decompensated patients. Nowadays in more developed centres, FAST is more readily available. To diagnose bowel injury, the WBC and red blood cell ratio in the lavage fluid is compared to the ratio in peripheral blood [59]. This technique remains too sensitive; as such, the low positive predictive value results in unnecessary laparotomies. Much more specific is the assessment for biochemical markers in the lavage fluid such as alkaline phosphatase and amylase [60–62]. There are no large contemporary studies on DPL in our current area of modern CT scans and observation-based selective NOM. Diagnostic peritoneal lavage still holds some merit and can be a useful tool in haemodynamically compensated patients who are not clinically evaluable and have CT findings prompting the suspicion of bowel injury. In these patients markers specific for bowel injury in the lavage fluid are assessed (and not the presence of red blood cells) [63]. When the test is negative, the likelihood of bowel injury is very low and as a result the patient will be spared a laparotomy. In the era of diagnostic laparoscopy, sending a sample of peritoneal fluid to the laboratory following a negative laparoscopy is highly recommended. The fluid analysis is logistically simple, inexpensive, and reassuring with no added risk to the patient [64, 65].

### Screening, diagnostic and therapeutic laparoscopy

Laparoscopy is less invasive than a laparotomy in both diagnostic and therapeutic settings [66, 67]. Laparoscopy compares positively against laparotomy in wound infections, adhesions, incisional hernias, and hospital stay [68, 69]. Arguments against laparoscopy highlight increased operative time, increased difficulties, need for senior supervision and a higher risk of missed injuries in less experienced hands.

In haemodynamically compensated patients with penetrating trauma, either LWE or a screening laparoscopy is routinely used to confirm peritoneal breach prompting further exploration by either diagnostic laparoscopy or explorative laparotomy [70, 71]. As mentioned above, diagnostic laparoscopy in the hands of a trained specialist can be extremely safe with some specific injuries, such as those involving the diaphragm, more conveniently diagnosed and treated laparoscopically [70]. Rates of missed injuries with diagnostic laparoscopy vary from 22 to 45% [72]. More recently, one centre’s 10-year experience with laparoscopic management for blunt trauma involved 131 patients and missed only one injury [73]. Additionally in a large meta-analyses of 3,362 laparoscopies, clinically relevant injuries were missed only twice [74]. As technology develops, image quality improves, and surgeons become more experienced with diagnostic laparoscopy in trauma scenarios, these rates can only further decrease [75–77]. However, until these rates decrease, a negative laparoscopy cannot entirely exclude bowel injury and clinical suspicion should still follow.

Therapeutic laparoscopy although more technically challenging, is a promising option [70]. According to a meta-analysis of eight observational studies and one randomized clinical trial (RCT), therapeutic laparoscopy reduces wound infections and pneumonia rates [74]. Therapeutic laparoscopy also may require less operative time and length of hospital stay in both penetrating and blunt trauma [66, 74, 78–81]. Hajibandeh reported average operating times of 52 versus 80 min for laparoscopy versus laparotomy in penetrating bowel injury (p = 0.0003) [74]. Length of hospital stay reported by three different comparative studies varied from 11-, 11- and 3-days respectively after therapeutic laparoscopy and compared favourably to laparotomy with 17-, 21- and 8-days (p = 0.004, p < 0.001 and p = 0.038) [67, 79, 80]. Despite the plethora of evidence supporting laparoscopy this remains an option only for the haemodynamically compensated trauma patients. Common repairs outlined by Di Saverio et al. include those of small bowel, mesentery, and colon. Small bowel perforations can be repaired via double layer suturing, larger defects may require resection and anastomosis either intracorporeally or extracorporeally. New haemostatic agents may assist in mesenteric vascular injury repair during laparoscopy [76]. Others have described laparoscopic Hartmann’s resections in detail, which may be suitable in traumatic colon injuries [82].

Those who are decompensated or have septic peritonitis benefit from laparotomy. Some clinicians in more recent years have begun pushing this boundary, expanding the criteria of patients suitable for laparoscopic intervention in complex trauma scenarios. Di Saverio et al. reports excellent outcomes in managing, laparoscopically, patients with previously considered absolute contraindications (such as diffuse peritonitis, impalement, and serious intra-abdominal adhesions) [76]. These considerations must be taken with caution, and due to their controversial nature, must only be considered by experienced laparoscopic surgeons. Nevertheless, laparotomy remains a safe and always available default surgical option. When comparing outcomes of laparotomy versus laparoscopy in the trauma setting, the expected outcome of a laparotomy is consistently worse, but the obvious selection bias needs to considered [83].

**Recommendations**

18. Diagnostic peritoneal lavage has a limited role. It can be used as an adjunct to a negative laparoscopy to definitively exclude bowel injury, particularly in conjunction with the use of biomarkers. **(GRADE: Moderate)**
19. Diagnostic laparoscopy can be used in haemodynamically compensated patients with highly sensitive findings of bowel injury on CT. **(GRADE: Moderate)**
20. In penetrating trauma, local wound exploration is used to confirm peritoneal breaching. When positive, serial clinical examinations should follow, where there is clinical suspicion for bowel injury a diagnostic/therapeutic laparoscopy or laparotomy is warranted. Conversion to laparotomy is always possible and highly recommended if any doubts or difficulties arise. **(GRADE: Moderate)**
21. Based on the surgeon experience and logistics of the trauma centre, bowel injuries identified during diagnostic laparoscopy can be treated laparoscopically. **(GRADE: Moderate)**

## Surgical options for bowel trauma

When faced with bowel injuries management options include (1) primary repair, (2) bowel resection with or without anastomosis, or (3) stoma (either at the site of injury or proximally) [84]. A primary anastomosis is performed at the time of initial laparotomy, whereas a delayed primary anastomosis is one that is usually performed at the time of the relook laparotomy, usually within 48–72 h, but can be performed on the third or fourth re-look if required.

### Primary repair

Bowel injuries should, where feasible, be managed by primary repair. Contraindications include destructive injuries with > 50% disruption of the bowel circumference, and mesenteric devascularisation with bowel ischaemia [85].

### Anastomosis

Small bowel continuity is preferable to diversion, however, the occurrence of an anastomotic leak in a trauma patients is linked to a steep increase in mortality (46% versus 1% in patients with or without an anastomotic leak, respectively; p < 0.001) [86]. The risks must be carefully weighted. Over the last ten years, published leak rates for colonic anastomosis ranged between 2 and 25% (Table 4). More distal anastomoses are complicated by higher leak rates (17%, 25% and 50% for right, transverse, and descending colon, respectively). Leak rates seem to be higher for delayed primary anastomoses when compared to primary anastomoses (18% *versus* 10%; p = 0.2) [87]. Additionally, anastomotic leak rates are higher in open abdomens (as negative pressure therapy can affect anastomosis healing) (6% *versus* 27% in closed *versus* open abdomens, respectively; p < 0.002) [88]. Delays to fascial closure of more than five days result in a greater likelihood of an anastomotic leak (18% versus 2%; p = 0.003) [89]. Similarly other authors have reported an eight-fold increase in anastomotic leak rate when the abdomen remains open after the first relook laparotomy (demonstrating anastomotic leak rates of 2%, 2% and 19% at first laparotomy, first relook and second relook, respectively; p < 0.001) [90].

**Table 4:** Reported anastomotic leak rates in trauma patients (2011–2021)

Authors, Year	Anastomotic site	Anastomoses: n	Anastomotic leaks: n (%)
Saar [122]	Colon	169	4 (2)
Sharpe [123]	Colon	44	2 (5)
Schnüriger [96]	Right colon	31	2 (17)
	Transverse colon	17	3 (25)
	Left colon	40	6 (50)
	Rectum	2	0
	Multiple	2	1 (8)
	Total	92	12 (13)
Ott [88]	Colon	116	16 (14)
Sharpe [124]	Colon	44	3 (7)
Anjaria [90]	Colon	83	10 (12)
	Small bowel	62	2 (3)
Burlew [92]	Right colon	38	1 (3)
	Transverse colon	5	1 (20)
	Left colon	22	10 (45)
	Total	127	14 **(**11**)**
Georgoff [89]	Colon	38	6 (16)
Oosthuizen [125]	Colon	20	5 (25)

The greater resilience and healing ability of the small bowel favours primary anastomosis in all settings. The few studies that have reported outcomes of small bowel anastomosis in trauma settings have reported leak rates of about 3% in both the primary and delayed primary anastomosis patients [91–93]. The use of indocyanine green fluorescent intraoperatively in trauma settings to predict anastomotic leak is a very new concept. Little data exists with no large prospective studies; however, case series appear promising and demonstrate a usefulness in assessing bowel integrity, an important prognostic factor in anastomotic leakage [94].

In summary, leaks and complications are higher when the anastomosis is performed after the first relook laparotomy, especially more than 48 h after the initial injury, or if abdominal fascial closure cannot be achieved at the time of the first relook laparotomy [95]. The presence and extent of other injuries, the haemodynamic status, degree of peritoneal contamination, and ongoing inotropic/blood product requirements are highly relevant factors in deciding between anastomosis or stoma. Other factors to consider include suboptimal resuscitation or reperfusion-related bowel wall oedema [96], time to surgery, and single anti-microbial use [97].

### Damage control surgery (DCS)

Some have advocated for the role of primary anastomosis during DCS [98], as it enables the surgeon to further inspect the anastamosis during relook laparotomy. More often, in the interest of time during a DCS laparotomy an anastomosis is not performed, and the bowel ends are stapled or closed rapidly with sutures [99, 100]. As reported above, a delayed primary anastomosis at relook laparotomy is burdened by an increased but acceptable leak rate (when the abdomen is closed) and it is advocated when the physiology has been completely restored and the patient is able to tolerate the burden of a possible anastomotic leak [101].

### Stoma

Diverting stomas are a crucial consideration in the presence of multiple colonic or sigmoid anastomoses. Loop stomas are preferred because their reversal is easier and associated with lower morbidity [92, 102]. Despite the low risk, stoma reversal remains far from uncomplicated and its risks should always be taken into consideration [103]. End stomas and Hartmann’s procedures are however safe when used to manage colon injuries. They should be highly considered in patients at high risk for a leak or leak-related morbidity [104, 105].

**Recommendations**

22. Primary repair of small bowel injuries is preferred when possible. **(GRADE: High)**
23. Primary anastomosis of colon injuries is safe in a subgroup of patients selected based on physiology, concomitant injuries, and resilience to a possible anastomotic leak. **(GRADE: Moderate)**
24. Diverting stomas remain a safe option and are recommended in high-risk patients with high-risk colon anastomoses. **(GRADE: Moderate)**
25. The risk of anastomotic leak following DCS increases with: **(GRADE: Moderate)**
  - Time from initial surgery
  - Ongoing transfusion requirements, ongoing inotropic support, tissue oedema and intraabdominal sepsis
  - Time to abdominal fascia closure

## Hand sawn versus staple anastomosis in bowel injuries

Emergency surgery particularly in the setting of trauma, differs from elective surgery in terms of pathology, perioperative physiology, and general management principles. There is particular concern that oedematous bowel (following splanchnic hypoperfusion and subsequent reperfusion in addition to the inflammatory host response) is less suited to staples rather than handsewn anastomosis [106]. The literature does not support the general feeling that trauma patients with bowel injuries requiring anastomosis who are managed with stapling have a higher rate of complications than those treated with a hand-sewn anastomosis. Some highlight increased bleeding complications when stapled, this is an observation not well reported in the literature. Although researchers have tried to address this hypothesis with cohort studies, no definitive RCT has been produced so far. Table 5 highlights the main findings of the few studies identified over the last two decades in the English literature. A multicentre prospective analysis of 207 patients with penetrating colonic injury found no difference in leak rates or abscess formation between the two methods of colonic anastomosis (6.3% in the stapled group and 7.8% in the handsewn group, p = 0.69) [107]. However, the Western Trauma Association conducted a multicentre retrospective cohort study of 199 trauma patients from five level 1 trauma centres, reported stapled anastomosis to be associated with a higher leak rate and abscess formation compared to the handsewn technique (4% in stapled group and 0% in the handsewn group, p = 0.04) [108]. Finally, Witzke [109] reported no leaks after 254 small bowel resections and anastomosis irrespective of the technique (supporting the well-known resilience of this organ) [91–93]. The difference in the anastomotic leak rate between the two techniques in the trauma patients is small or non-existent. Similar differences exist between single- and double-layer suturing techniques [110].

**Table 5:** Leak rates following handsewn or stapled anastomosis in trauma patients

Study	Anastomosis in trauma patients (n)	Location/mechanism	Leak (n)/handsewn (n) (%)	Leak (n)/stapled (n) (%)	*P*
Brundage [108]	289	SB and colon /BAT and PAT	0/114 (0)	7/175 (4)	0.04
Demetriades [107]	207	Colon/PAT	11/128 (8%)	4/79 (6%)	0.3
Witzke [109]	254	SB/BAT and PAT	0/145	0/79	ns
Kirkpatrick [106]	127	SB	1/38	3/64	ns

*BAT* blunt abdominal trauma, *PT* penetrating abdominal trauma, *SB* small bowel

These findings mimic those on (non-trauma) emergency surgery patients. Even in this group of patients, despite a larger number of studies and one RCT, no consistent outcome advantage exists among the two different techniques exists. An RCT of 201 patients undergoing emergent bowel resection compared the two techniques and showed minimal, and not statistically significant differences, differences in leak rates (6.6% versus 5.2%) [111]. A retrospective cohort study of 231 similar patients reported a higher anastomotic failure rate with stapled technique compared than handsewn (15% versus 6.1%, p = 0.03) [112]. Clinically irrelevant longer operating times in the handsewn anastomosis group were also shown (205 versus 193 min, p = 0.02). More recently, a multicentre prospective study sponsored by the American Association for the Surgery of Trauma on 595 patients and 649 bowel anastomoses for (non-trauma) emergent bowel resection (253 handsewn and 396 stapled) was completed. They reported a higher but not statistically significant leak rate in handsewn group (15.4% handsewn versus 10.6% stapled group, p = 0.07). The operative time was equivalent (165 versus 152 min for handsewn versus stapled group, respectively) [113]. The anastomotic leakage rates may have been irregularly high, as more patients received anastomosis involving large bowel to large bowel, which tends to have higher leakage rates when compared to small bowel [113]. Finally, a systematic review and meta-analysis of 1120 emergency general surgery patients from 7 studies (5 trauma studies, 2 emergency general surgery studies) showed, using the random effects model, no differences between the handsewn and stapled groups in anastomotic failure, abscess formation, fistula, duration of hospital stays, or mortality between these groups [110].

Patient selection and operator surgical skills bias all retrospective studies, which cannot be corrected even by the most advanced statistical methods. It is highly likely that the most senior surgeon treats the most unwell patients, who in general will tend to perform a handsewn anastomosis. It will be interesting to observe outcomes in the decade to come, now that even the senior surgeon cohort “has grown up with staples in their hands”. Again, a multicentre RCT would be an ideal tool to overcome these biases and address these controversies. Meanwhile, surgeons will continue to be guided by their insight and previous experience rather than prescriptive recommendations.

**Recommendations**

26. There is a lack of evidence to demonstrate the superiority of anastomotic techniques following a bowel resection in trauma patients. **(GRADE: High)**
27. The decision to perform either a handsewn or stapled bowel anastomosis in the setting of emergency trauma laparotomy should be individualised to the patient’s condition and the surgeon’s technical abilities. **(GRADE: Moderate)**

## Missed bowel injury: management and outcome

The diagnosis of small bowel and colonic injuries, particularly in blunt trauma, can be challenging, and a delay in diagnosis can be catastrophic, with a steep increase in morbidity and mortality. Small bowel and colonic full-thickness injuries may take a longer time to produce significant signs and symptoms due to the relatively neutral pH of small bowel content and the retroperitoneal position of portions of the duodenum and colon which could hinder the development of classic peritonitis. To compound the difficulties, clinical examination of multisystem trauma patients is unreliable due to distracting injuries, pain medications, sedation and/or a comatose state [106, 114]. Furthermore, NOM of solid organ injury is routinely done nowadays, which is linked to delays in diagnosis of bowel injury. Given this scenario, serial physical examinations, serial CT scans and a high index of suspicion have become crucial to avoid missing hollow viscus injuries [115].

A retrospective cohort study of 52 patients with delayed bowel repair of more than 24 h showed no significant difference in hospital and ICU length of stay, morbidity, and mortality (but an association with bowel resection rather than primary repair was found in the delayed group) [115]. Similarly, on 90 trauma patients with small bowel injuries, a delay to surgery of more than 6 h did not lead to a statistically significant difference in morbidity (51.6% and 53.6%, p = 0.29) and mortality (16.1% and 28.6%, p = 0.28) [116].

Larger studies, instead, have shown delays to surgical treatment to be associated with increased morbidity and mortality. A multicentre cohort study of 198 patients with small bowel injuries following blunt trauma reported a statistically significant correlation between mortality rates and increasing time interval to surgical intervention (2%, 9%, 17%, and 31% for time-to-surgery groups of < 8, 8–16, 16–24 and > 24 h, respectively; p = 0.009) [117]. Similarly, in a cohort of 195 blunt trauma patients with hollow viscus injury, a delay of more than 5 h between admission and laparotomy was linked to an increased risk of mortality [118]. One more cohort of 62 blunt trauma patients with bowel injury showed an association between a delay of more than 8 h to surgical treatment and higher rates of serious complications (27% vs 61%; p < 0.01) and sepsis (16% vs 28%; p = 0.03) [1]. The findings of these studies highlight the importance of prompt decision making in all trauma situations, whether a patient arrives immediately to hospital following injury or there is a delay to presentation. These controversial findings and the lack of larger studies have resulted in a recent meta-analysis against an association between delay to bowel injury repair and increased mortality [5]. Many of the above-mentioned studies do not exclude severe traumatic brain injury patients (in whom bowel injuries are often missed). It is plausible that some of the mortalities were associated to brain injury rather the delay to surgery itself.

Mesenteric injury is a potentially life-threatening injury following blunt abdominal trauma and can result in severe haemorrhage and/or bowel ischemia. The clinical presentation is the direct consequence of progressive ischemia from a mesenteric tear or hematoma and varies from mucosal ulceration, strictures, obstruction, and ultimately perforation. Occasionally these can remain subclinical upon presentation following a delayed period ranging from two weeks to three months. Therefore, patients at risk of such injuries need to be followed-up closely for several weeks [119–121].

**Recommendations**

28. In the context of blunt abdominal trauma with or without solid organ injury, bowel injuries are often missed. A high index of suspicion is required. **(GRADE: High)**
29. Delay in the diagnosis of bowel injury is linked to increased morbidity and mortality. **(GRADE: Moderate)**
30. Long-term follow-up of patients with blunt abdominal trauma is required to identify the sequelae of mesenteric injuries. **(GRADE: Low)**

## Conclusion

Several knowledge gaps in the literature have been identified and highlighted by this collaborative attempt to guide clinicians with evidence-based recommendations. A summary table of the recommendations can be found below (Table 6). The importance and utility of these recommendations will be demonstrated through clinical practice in coming years. With the gaps identified in the literature, we envision a collaborative effort to assess anastomosis techniques with a multicentre RCT, validate the CT findings of bowel injury in trauma in prospective multicentre studies, and refine nonoperative management requirements for penetrating abdominal trauma. Future research should focus on minimizing missed bowel injuries, combining clinician awareness and improved understanding of the utility of biomarkers in a trauma setting.

**Table 6:** Summary of recommendations

Recommendation	Grade
Management of the awake and oriented blunt abdominal trauma patient starts with the primary survey, E-FAST, physical examination and the secondary survey, blood chemistry, vital signs followed by contrast-enhanced abdominal CT	High
The presence of a seatbelt sign should prompt a CT scan and a high index of suspicion for bowel injury	High
Patients with high-risk mechanisms (i.e. handlebar, seatbelt sign) and non-specific CT findings should be admitted for observation including serial clinical examination	Moderate
In patients not clinically evaluable, the diagnosis of hollow viscus injuries relies on injury pattern, vital signs, inflammatory markers trends and follow-up CT	Moderate
In selected cases a repeat CT might be considered. Patients with equivocal signs on initial CT scan should be re-imaged after 6 h. Patients that demonstrate evolving clinical signs suspicious for bowel injury, re-imaging should be considered	High
Although highly sensitive, serum procalcitonin and CRP are not necessarily specific and as supportive biomarkers will help to exclude bowel injuries; but if too heavily relied upon, may lead to nontherapeutic laparotomy, or missed bowel injury	Moderate
The presence of highly specific CT findings such as extraluminal air, extraluminal oral contrast, or bowel-wall defects warrants prompt surgical exploration	Moderate
The presence of highly sensitive CT findings such as free fluid in the absence of solid organ injury, abnormal enhancement of bowel wall, and mesenteric stranding can be used as an adjunct to the clinical picture but should not solely determine management	Moderate
Scoring systems that include radiologic, biochemical, and clinical signs can guide management in difficult scenarios	Moderate
A repeat CT scan can be considered in patients with high-risk mechanisms without peritoneal signs and subtle signs on initial CT of bowel injury who do not show clinical improvement or are not clinically evaluable	Moderate
NOM can be performed at specialised centres in patients with penetrating abdominal trauma provided that the patient is haemodynamically compensated and cooperative. NOM might be more suitable for stab wounds vs GSW	Moderate
When CT does not identify hard signs of bowel injury, LWE or screening laparoscopy to investigate for peritoneal violation will guide toward a laparotomy or NOM. Patients without peritoneal violation can be safely discharged	Moderate
NOM requires at least 48 h of serial clinical examinations, performed by consistent specialists or consultants, vital sign monitoring, and serial inflammatory markers testing	Moderate
Following penetrating trauma, highly specific CT findings for bowel injury following penetrating trauma include extraluminal air, extraluminal contrast, bowel-wall defects and metallic fragments within the intestinal wall or lumen	Moderate
Following penetrating trauma, highly sensitive CT findings for bowel injury following penetrating trauma include free fluid in the absence of solid organ injury, abnormal enhancement of bowel wall and mesenteric stranding. These can be used as an adjunct in the clinical picture but should not solely determine management	Moderate
IV contrast-enhancing CT scan has equal sensitivity to triple contrast in detecting bowel injury and is favourable in time-sensitive trauma situations	Low
Serial clinical examinations are complementary to CT in guiding surgical management in trauma centres that practice the NOM approach in penetrating abdominal trauma	Moderate
Diagnostic peritoneal lavage has a limited role. It can be used as an adjunct to a negative laparoscopy to definitively exclude bowel injury, particularly in conjunction with the use of biomarkers	Moderate
Diagnostic laparoscopy can be used in haemodynamically compensated patients with highly sensitive findings of bowel injury on CT	Moderate
In penetrating trauma, local wound exploration is used to confirm peritoneal breaching. When positive, serial clinical examinations should follow, where there is clinical suspicion for bowel injury a diagnostic/therapeutic laparoscopy or laparotomy is warranted. Conversion to laparotomy is always possible and highly recommended if any doubts or difficulties arise	Moderate
Based on the surgeon experience and logistics of the trauma centre, bowel injuries identified during diagnostic laparoscopy can be treated laparoscopically	Moderate
Primary repair of small bowel injuries is preferred when possible	High
Primary anastomosis of colon injuries is safe in a subgroup of patients selected based on physiology, concomitant injuries, and resilience to a possible anastomotic leak	Moderate
Diverting stomas remain a safe option and are recommended in high-risk patients with high-risk colon anastomoses	Moderate
The risk of anastomotic leak following DCS increases with: Time from initial surgery Ongoing transfusion requirements, ongoing inotropic support, tissue oedema and intraabdominal sepsis Time to abdominal fascia closure	Moderate
There is a lack of evidence to demonstrate the superiority of anastomotic techniques following a bowel resection in trauma patients	High
The decision to perform either a handsewn or stapled bowel anastomosis in the setting of emergency trauma laparotomy should be individualised to the patient’s condition and the surgeon’s technical abilities	Moderate
In the context of blunt abdominal trauma with or without solid organ injury, bowel injuries are often missed. A high indexed of suspicion is required	High
Delay in the diagnosis of bowel injury is linked to increased morbidity and mortality	Moderate
Long-term follow-up of patients with blunt abdominal trauma is required to identify the sequelae of mesenteric injuries	Low

## Data Availability

All data was obtained through focused literature searches involving both Medline and PubMed online databases. No new data was obtained in the creation of this paper.

## Abbreviations

BAT: Blunt abdominal trauma
PAT: Penetrating abdominal trauma
SB: Small bowel
CT: Computed tomography
DPL: Diagnostic peritoneal lavage
DCS: Damage control surgery
FAST: Focused Assessment with Sonography for Trauma
LWE: Local wound exploration
MVA: Motor Vehicle Accident
NOM: Non-operative management
qSOFA: Quick sequential organ failure assessment
WCC: White cell count
